# Antioxidant Capacity of “Mexican Arnica” *Heterotheca inuloides* Cass Natural Products and Some Derivatives: Their Anti-Inflammatory Evaluation and Effect on *C. elegans* Life Span

**DOI:** 10.1155/2015/843237

**Published:** 2015-03-02

**Authors:** José Luis Rodríguez-Chávez, Elvia Coballase-Urrutia, Antonio Nieto-Camacho, Guillermo Delgado-Lamas

**Affiliations:** ^1^Instituto de Química, Universidad Nacional Autónoma de México, Circuito Exterior, Ciudad Universitaria, 04510 México, DF, Mexico; ^2^Laboratorio de Neuroquímica, Instituto Nacional de Pediatría, Insurgentes Sur 3700-C, 04530 México, DF, Mexico

## Abstract

It has been suggested that the accumulation of biomolecular damage caused by reactive oxygen species (ROS) contributes to aging. The antioxidant activity is related to the ability of certain compounds to protect against the potentially harmful effect of processes or reactions involving ROS. This ability is associated with the termination of free radical propagation in biological systems. From *Heterotheca inuloides* various compounds which have shown to possess antioxidant capacity and scavenging ROS. The aim of this study was to determine the antioxidant capacity of additional natural components isolated from *H. inuloides* and some semisynthetic derivatives, their anti-inflammatory activity and the effect on *Caenorhabditis elegans* nematode life span. Compounds showed ability to inhibit various biological processes such as lipid peroxidation, scavenge nonbiological important oxidants such as ^1^O_2_, OH^∙^, H_2_O_2_, and HOCl and scavenge non biological stable free radicals (DPPH). Some cadinane type compounds showed possess antioxidant, ROS scavenging capacity, anti-inflammatory activity, and effect on the *C. elegans* life span. Flavonoid type compounds increased the life of the nematode and quercetin was identified as the compound with the greatest activity. The modification of chemical structure led to a change in the antioxidant capacity, the anti-inflammatory activity, and the survival of the worm.

## 1. Introduction

Reactive oxygen species (ROS) exist as products of normal cellular physiology and play vital roles in the stimulation of signaling pathways in plant and animal cells [1]. Aerobic organisms produce ROS during the reduction of molecular oxygen by mitochondria [2]. ROS include free radicals such as superoxide anion (O_2_^−^), hydroxyl radical (OH^∙^), and nonradical molecules like hydrogen peroxide (H_2_O_2_), singlet oxygen (^1^O_2_), and other species such as nitric oxide (NO), hypochlorous acid (HOCl), and peroxynitrite (ONOO^−^). When the level of ROS exceeds away cellular factors responsible for protecting cellular biomolecules against damage generated by oxidizing species is said to be in a state of “oxidative stress.” Under these conditions ROS can damage biomolecules like nucleic acids, proteins, lipids, carbohydrates, and enzymes. This condition has been implicated in the pathogenesis of a number of multiple pathologies such as senescence [3], ischemia/reperfusion injury [4], neurodegenerative diseases [5], infectious processes [6], rheumatoid arthritis [7], arterial diseases [8], obesity, diabetes, chronic kidney disease [9], and other ailments.

*Heterotheca inuloides* is commonly known as “Mexican arnica” and it is known by other names in different regions of Mexico [10, 11]. In Mexican traditional medicine the infusions of this plant are mainly used for treatment of contusions and bruises [12]. Phytochemical studies of this plant, allowed isolating different classes of compounds, mainly cadinane type sesquiterpenes [13], flavonoids [14], and phytosterols [15]. Previous studies have reported that metanolic extract and certain natural products isolated from* H. inuloides* dried flowers have antioxidant activity and ability to inhibit lipid peroxidation, scavenging ROS and act as hepatoprotective agents [16–18]. However, the antioxidant activity of natural products isolated from the acetone extract has not been reported. In the present study, we evaluated the antioxidant and ROS scavenging capacity of* H. inuloides* metabolites isolated from the acetonic extract and prepared semisynthetic derivatives and also evaluated their anti-inflammatory effects and effect on the* C. elegans* life span. Here we report the results considering the structure-activity relationships.

## 2. Materials and Methods

### 2.1. Reagents

All reagents used were of analytical grade. Sodium pyruvate, dimethyl thiourea (DMTU), nordihydroguaiaretic acid (NDGA), ascorbic acid, histidine, xylenol orange (FOX), 2,2-diphenyl-1-picrylhydrazyl (DPPH), dimethylsulfoxide (DMSO), N,N-dimethyl-4-nitrosoaniline (DMNA), catalase, xanthine, xanthine oxidase, nitroblue tetrazolium (NBT), dL-penicillamine 2-thiobarbituric acid (TBA), *α*-tocopherol, Folin and Ciocalteu's phenol reagent, 3,5-di-tert-4-butylhydroxytoluene (BHT), and 5-fluoro-2′-deoxyuridine were purchased from Sigma-Aldrich (Toluca, Mexico, or Sigma, St. Louis, MO). Absolute ethanol, hydrogen peroxide (H_2_O_2_), sulfuric acid (H_2_SO_4_), methanol, ethylenediaminetetraacetic acid disodium salt (EDTA), NaCl, KH_2_PO_4_, NaHPO_4_·7H_2_O, NaOH, and H_2_SO_4_ were purchased from Merck-Mexico. Sodium hypochlorite (NaOCl) was purchased from Hycel (Mexico City, Mexico).

### 2.2. Strains of* C. elegans*

Experimental* C. elegans* strain was wild type N2 (Bristol). The strain was obtained from the Caenorhabditis Genetics Center (University of Minnesota, Minneapolis, MN, USA) and was maintained on nematode growth medium (NGM) at 20°C as described previously by Brenner [19]. Age-synchronized worms were generated in all experiments through the sodium hypochlorite method. Worms were allowed to hatch in Petri dishes in liquid* S*-medium with concentrated* Escherichia coli* OP50 as food [20].

### 2.3. Plant Material

Flowers of* H. inuloides* (provided by MIXIM Laboratories, Naucalpan, Mexico) were collected in 2010, in the town of Mesas Altas de San Juan Xoconusco, municipality of Donato Guerra (State of Mexico), and authenticated by MS Abigail Aguilar-Contreras. A voucher of plant material was deposited under code IMSSM-16064 in the Medicinal Plant Herbarium of the Instituto Mexicano del Seguro Social (IMSS, Mexico City).

### 2.4. *Heterotheca inuloides* Metabolites and Derivatives

**Figure 1:**
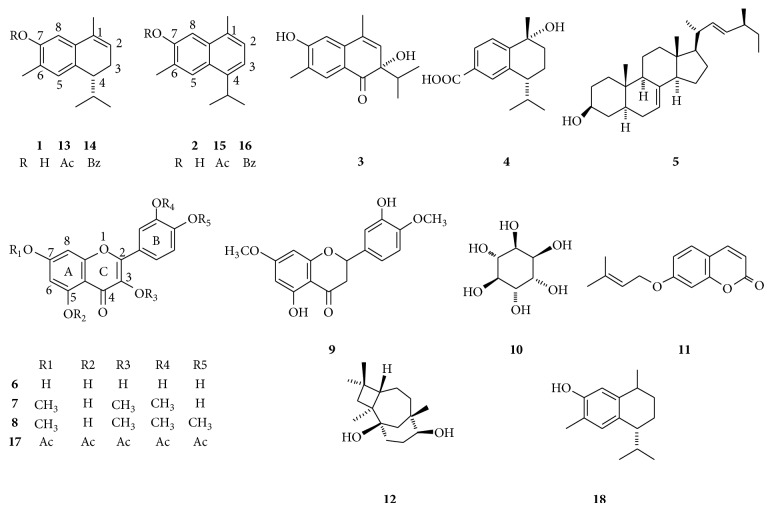
Natural products isolated from* H. inuloides* flowers and derivatives.

Compounds** 1–12** were isolated from the acetone extract of dried flowers of* H. inuloides* in a previous study. Semisynthetic compounds 7-acetoxy-3,4-dihydrocadalene (**13**), 7-benzoxy-3,4-dihydrocadalene (**14**), 7-acetoxycadalene (**15**), 7-benzoxycadalene (**16**), quercetin pentaacetate (**17**), and 7-hydroxy calamenene (**18**) were obtained by conventional chemical procedures as was previously described [21] (Figure 1). Due to the paucity of material compounds** 10** and** 11** were not included in all the bioassays.

### 2.5. Antioxidant Effects and ROS Scavenging of* H. inuloides* Metabolites

#### 2.5.1. Estimation of Lipid Peroxidation

Lipid peroxidation was measured by TBARS assay using rat brain homogenates [22], with some modifications. Adult male Wistar rats (200–250 g) were provided by the Instituto de Fisiología Celular, UNAM, and their use was approved by the Animal Care and Use Committee [23]. The animals sacrifice was carried out avoiding unnecessary pain with CO_2_, cerebral tissue (whole brain) was rapidly dissected and homogenized. The homogenate was centrifuged for 10 min at 3400 rpm to yield a pellet that was discarded; protein content in the supernatant was measured using the Folin and Ciocalteu's phenol reagent [24] and adjusted to 2.66 mg protein/mL with PBS. The supernatant (375 *μ*L) was incubated at 37°C for 30 min in presence of test sample (25 *μ*L) dissolved in DMSO or ethanol and 50 *μ*L of EDTA solution (20 *μ*M). Lipid peroxidation was started adding 50 *μ*L of a freshly prepared 100 *μ*M FeSO_4_ solution (final concentration 10 *μ*M) and incubated at 37°C for 60 min TBARS were determined as described by Ohkawa et al. [25] with some modifications. Concentration of TBARS was calculated by interpolation in a standard curve of tetrametoxipropane (TMP) [26]. Final results were expressed as mmoles of TBARS per mg of protein. The inhibition ratio (%) was calculated using the following formula:

(1) $$\begin{array}{c} \text{Inhibition}\;\;\text{ratio}\;\;\left(\%\right)=\frac{\left(C-E\right)}{C}\times 100\%, \end{array}$$

where *C* was the absorbance of control and *E* was the absorbance of the test sample.

#### 2.5.2. DPPH^∙^ Scavenging Capacity

DPPH assay was assessed in 96-well microtiter plates according to the method of Blois [27] with minor changes. Briefly, 0.05 mL of test compounds in DMSO at different concentrations was added to an ethanolic solution of 2,2-diphenylpicrylhydrazyl (DPPH^∙^) (133.33 *μ*M, 0.150 mL). The control sample contained distilled water. Reaction mixtures were incubated at 37°C for 30 min in the dark. After incubation the absorbance was measured at 515 nm in a microplate reader ELx808 (BioTek Instruments, Inc., Vermont, USA). The % inhibition of each compound was determined by comparison with a DPPH ethanol blank solution [28]. The scavenging capacity is given as percent (%) DPPH scavenged, calculated as [(optical density of control − optical density of compound)/(optical density of control) × 100]; *α*-tocopherol was used as standard.

#### 2.5.3. Superoxide Radical-Scavenging Activity

The superoxide radicals (O_2_^−^) were generated through the system xanthine oxidase [29]. O_2_^−^ in the assay system and xanthine oxidase activity were measured as NBT reduction using a DU-640 series Beckman spectrophotometer. 800 *μ*L of the following reaction mixtures: 90 *μ*M xanthine, 16 mM Na_2_CO_3_, 22.8 *μ*M NBT, and 18 mM phosphate buffer (pH 7.0) was mixed with 100 *μ*L of different concentrations of* H. inuloides* metabolites. The reaction was started by the addition of 100 *μ*L of xanthine oxidase (168 U/L). Optical density was registered both at 295 (for uric acid production) and at 560 nm (for O_2_^−^ in the assay system). The absorbance was determined using a Beckman DU640 Spectrophotometer (Beckman Coulter, Inc. California, USA).

#### 2.5.4. Peroxide Scavenging Activity

Determination of scavenging capacity was made by ferrous ion oxidation-xylenol orange (FOX) assay [30]. Concentration of H_2_O_2_ was calculated from a standard curve prepared with increasing H_2_O_2_ concentrations. A solution of 75 *μ*M H_2_O_2_ was mixed (1 : 1 v/v) with water (0% scavenging tube) or with different concentrations of* H. inuloides* metabolites and incubated for 30 min at room temperature. After this, H_2_O_2_ was measured by the following method. Briefly, 9 volumes of 4.4 mM BHT in HPLC-grade methanol were mixed with 1 volume of 1 mM xylenol orange and 2.56 mM ammonium ferrous sulphate in 0.25 M H_2_SO_4_ to give the working FOX reagent. 45 *μ*L of the metabolite solutions and 45 *μ*L of 75 *μ*M H_2_O_2_ were dispensed in 1.5 mL Eppendorf tubes and mixed with 10 *μ*L of HPLC-grade methanol immediately followed by the addition of 0.9 mL of FOX reagent, mixed on a vortex mixer for 5 s and then incubated at room temperature for 10 min. The tubes were centrifuged for 15,000 g for 10 min and absorbance at 560 nm was read against a methanol blank using a Beckman DU640 Spectrophotometer (Beckman Coulter, Inc. California, USA). The concentration of H_2_O_2_ was calculated from a standard curve prepared with increasing H_2_O_2_ concentrations. Sodium pyruvate was used as standard for H_2_O_2_ scavenging activity.

#### 2.5.5. Hydroxyl Radical-Scavenging Activity

The malondialdehyde formed from the decay of deoxyribose was evaluated in reaction with thiobarbituric acid and measured at 532 nm [31]. The reaction mixture containing deoxyribose (0.056 mM), H_2_O_2_ (1 mM), potassium phosphate buffer (10 mM, pH 7.4), FeCl_3_ (0.2 mM), EDTA (0.2 mM), ascorbic acid (0.2 mM), and 100 *μ*L of different concentrations of* H. inuloides* was incubated in a water bath at 37 ± 0.5°C for 1 h. The extent of the deoxyribose degradation by the OH^∙^ formed was measured by the thiobarbituric acid test at 532 nm using a Beckman DU640 Spectrophotometer (Beckman Coulter, Inc. California, USA). The ability of* H. inuloides* metabolites to scavenge OH^∙^ was compared with that of DMTU (0, 1, 2, 10, 20, and 31 *μ*g/mL). The final results were expressed as inhibition percent in relation to a control test (without the sample).

#### 2.5.6. Singlet Oxygen (^1^O_2_) Scavenging Activity

The ability of the compounds to scavenging ^1^O_2_ was detected spectrophotometrically at 440 nm using the bleaching of N,N-dimethyl p-nitrosoaniline (DMNA) as a specific detector [32]. The assay mixture contained 45 mM Na-phosphate buffer (pH 7.1), 10 mM histidine, 10 mM NaOCl, 10 mM H_2_O_2_, 50 *μ*M DMNA, and 0.1 mL of different concentration of compounds. The total volume of reaction (2.0 mL) was incubated at 30°C for 40 min. The extent of ^1^O_2_ production was determined by measuring the decrease in the absorbance of DMNA at 440 nm using a Beckman DU640 Spectrophotometer (Beckman Coulter, Inc. California, USA). The relative scavenging efficiency (% inhibition production of ^1^O_2_) of* H. inuloides* metabolites was estimated from the difference in absorbance of DMNA with and without the addition of compounds being tested or reference compound. Glutathione (0, 0.92, 1.53, 2.15, 2.45, and 3.07 mg/mL) was used as standard for ^1^O_2_ scavenging.

#### 2.5.7. HOCl Scavenging Assay

The HOCl scavenging activity was evaluated by measuring the decrease in absorbance of catalase at 404 nm and was carried out as described by Aruoma and Halliwell [33] with minor changes. Briefly, 150 *μ*L of 49.8 *μ*M bovine liver catalase solution (16.6 *μ*M, final concentration) was mixed with 150 *μ*L of 18 mM HOCl (6 mM, final concentration) and 150 *μ*L of* H. inuloides* metabolite solutions in increasing concentrations or the reference compound ascorbic acid (0, 22, 44, 88 and 176 *μ*g/mL). Spectra (370–450 nm) of catalase alone, catalase plus HOCl, or catalase plus HOCl and* H. inuloides* metabolites or the reference compound were registered and the optical densities (OD) at 404 nm were determined using a Beckman DU640 Spectrophotometer (Beckman Coulter, Inc., California, USA). The value of the OD of catalase alone minus the OD of catalase plus HOCl was considered as 100% of degradation of catalase (or 0% of scavenging activity), and the difference of the catalase alone minus the OD of the catalase plus HOCl in presence of either* H. inuloides* metabolites or reference compound was compared against this value. The ability of TPM to scavenge HOCl was compared with that of ascorbic acid.

### 2.6. Evaluation of Anti-Inflammatory Effect

The biological model employed was ear edema in mice induced by 13-ethyl-12-O-tetradecanoylphorbol (TPA), as described by Rao et al. [34] with slight modifications. CD1 male mice were used. Mice were administered with sodium pentobarbital (31.5 mg/kg, ip) and TPA solution (2.5 *μ*g/ear) dissolved in ethanol (10 *μ*L). This was done topically on the right ear, in both faces of the ear. The left ear received only ethanol, 10 min after the test substances (1 *μ*mol/ear) or indomethacin (0.31 *μ*mol/ear) as drug reference. The tested substances and indomethacin were dissolved in 20 *μ*L of acetone and administered on both sides of the ears (10 *μ*L/side). The control only received the vehicle (20 *μ*L of acetone). Four hours later the mice were killed with CO_2_. A 7 mm diameter plug was removed from each ear. The swelling was assessed as the difference in weight between right and left ear plugs. The anti-inflammatory activity was expressed as inhibition of edema (IE) in percent relative to the edema formed in control animals according with the following formula: IE (%) = 100 − [B × 100/A], where A = edema induced by TPA alone, and B = edema induced by TPA plus sample.

### 2.7. Lifespan Assay

Lifespan assays were assessed in liquid medium at 20°C in 96-well plates (Corning, NY) and were carried out according to the established protocols [35]. Briefly, the nematodes were age-synchronized and distributed in wells as L1 larvae (10–20 animals per well) together with* Escherichia coli* OP50. To prevent self-fertilization, 5-fluoro-2′-deoxyuridine was added 36 h after seeding (0.12 mM final). Media were supplemented with different doses of compounds (100 or 200 *μ*M). DMSO/ethanol (50/50% v/v) was included as a solvent control. The worms were monitored daily to observe the number of live worms in each treatment, the fraction of animals alive was scored on the basis of body movement. Observations and worm counts were performed using a microscope Eclipse TS 100 (Nikon Instruments Inc., Tokyo, Japan).

### 2.8. Statistical Analysis

For lipid peroxidation and DPPH scavenging data were represented as mean ± standard error of mean (SEM). Data were analyzed by one-way ANOVA followed by Dunnett's test for comparisons against control. Values of *P* ≤ 0.05 (∗) and *P* ≤ 0.01 (∗∗) were considered statistically significant. The inhibitory concentration 50 (IC_50_) was estimated by means of a linear regression equation. For scavenging capacity data were expressed as mean ± SEM. The data were compared against the blank tube without* H. inuloides* metabolites or the reference compounds, using one-way analysis of variance (ANOVA) followed by the Dunnett's Multiple Comparison test (GraphPad Prism 4.0 Software, San Diego, CA, USA). *P* < 0.05 was considered statistically significant. The scavenging capacity was expressed as the 50% inhibitory concentration (IC_50_) value, which denotes the concentration of* H. inuloides* metabolites or the reference compounds required to give a 50% reduction in oxidating effect relative to the blank tube. The data of the lifespan assays were processed using the GraphPad Prism 4.0 Software, (GraphPad Software, Inc., San Diego, CA, USA). Survival was plotted by the Kaplan-Meier method and the curves compared for significance using the log-rank test.

## 3. Results

### 3.1. Antioxidant Effects and ROS Scavenging

Our results showed that only compounds** 2**,** 6**, and** 9** displayed the ability to reduce DPPH radical with a IC_50_ of 30.66 ± 8.14 *μ*M, 13.11 ± 1.2 *μ*M, and 6.97 ± 0.14 *μ*M. Compounds** 1** and** 7** showed slight activity with IC_50_ higher than 100 *μ*M. The other compounds tested showed a rate of antioxidant capacity less than 25% during the preliminary screening and therefore were not considered active (Table 1).

In peroxidation assay some compounds showed ability to inhibit this process. The inhibitory capacity of these compounds decreased in the following order:** 18** >** 13** >** 2** >** 17** >** 6 **>** 1 **>** 7 **>** 9** >** 11**. Compound** 18** had an IC_50_ of 0.58 ± 0.008 *μ*M and showed to possess greater ability to inhibit lipid peroxidation than *α*-tocopherol used as reference substance whose IC_50_ was 6.78 ± 2.16 *μ*M. The derivatives** 14**,** 15**, and** 16** showed little ability to inhibit lipid peroxidation with IC_50_ higher than 100 *μ*M.

**Figure 2:**
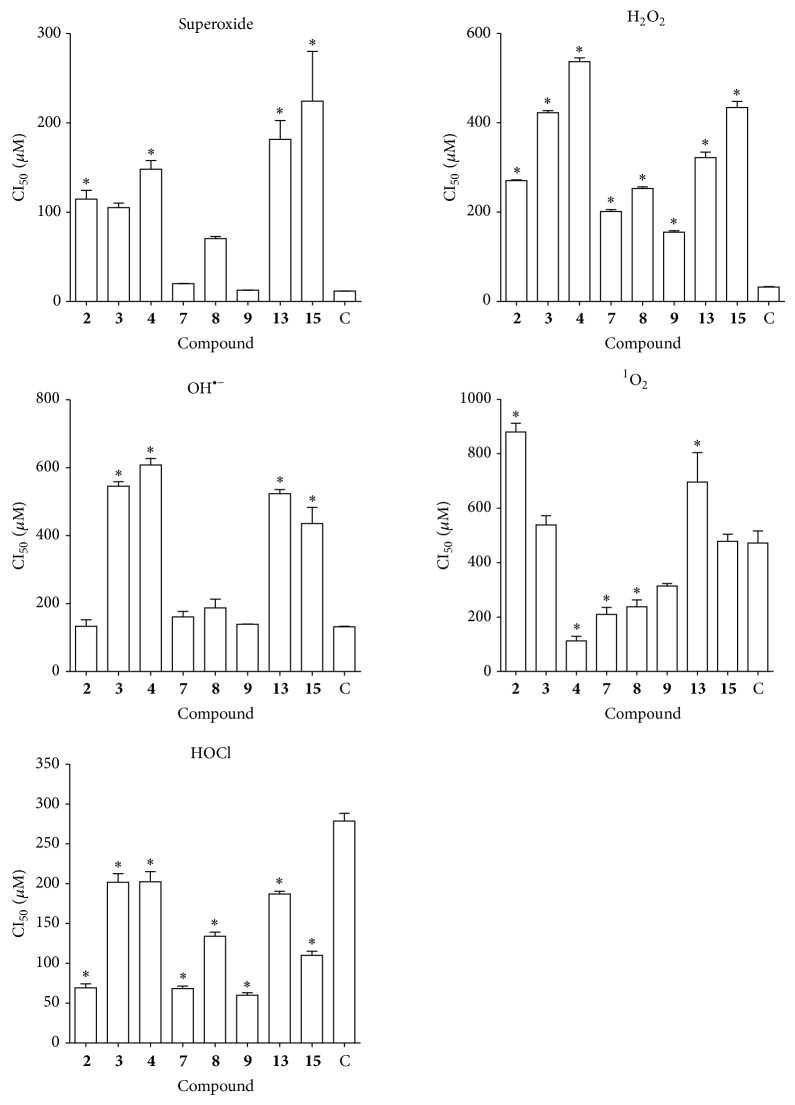
ROS scavenging capacity of* H. inuloides* metabolites and semisynthetic derivatives. C=NDGA (superoxide); pyruvate (H_2_O_2_); DMTU (OH^−^); GSH (^1^O_2_); ascorbic Ac. acid (HOCl). The results were analyzed by ANOVA. The Dunnett's Multiple Comparison test was used to compare outcomes between experimental and control group. ^*^ *P* < 0.05.

The* H. inuloides* natural products, derivatives as well as the reference compounds, displayed O_2_^−^, HOCl, H_2_O_2_, ^1^O_2_, and OH^∙^ scavenging activity in a concentration-dependent way. The IC_50_ values were calculated from the dose-response curve (Table 2, Figure 2).

The hypochlorous acid scavenging capability decreases in the following order** 9** >** 7** >** 2** >** 13** >** 15** >** 8** >** 3** >** 4**. Compound** 9** showed to possess the higher scavenging ability against HOCl. Compounds** 13** and** 15**, acetylated derivatives of compounds** 1** and** 2**, displayed a reduction in radical scavenging ability, with an increase in the IC_50_ value. The standard ascorbic acid showed an IC_50_ value of 278.6 ± 16.7 *μ*M.

In the case of hydroxyl radical, the capacity to scavenging decreased in the following order:** 2** >** 9** >** 7** >** 8** >** 15** >** 13** >** 3** >** 4**. The IC_50_ value for the dimethylthiourea used as reference substance was 131.5 ± 3.6 *μ*M and was lower than those of the compounds tested.

*H. inuloides* metabolites were capable of scavenging hydrogen peroxide in an amount-dependent manner. Results showed that the scavenging activity values decreased in the order of** 9** >** 7** >** 8** >** 2** >** 13** >** 3** >** 15** >** 4**. Compound** 9** showed the best scavenging ability with an IC_50_ of 154 ± 6.3 *μ*M; however, none of the compounds showed higher activity than the pyruvate used as reference substance whose IC_50_ was 32.2 ± 1.7 *μ*M.

The superoxide scavenger capacity decreased in the following order:** 9** >** 7** >** 8** >** 3** >** 2** >** 4** >** 13** >** 15**. Compound** 9** showed to possess the best ability to trap superoxide radical with IC_50_ value of 12.2 ± 0.22 *μ*M; however, it is not statistically different from NDGA reference compound whose IC_50_ was 11.6 ± 0.13 *μ*M.

With respect to the singlet oxygen scavenger capacity the ability of the compounds to trap the singlet oxygen decreased in the following order** 4** >** 7** >** 8** >** 9** >** 15** >** 3** >** 13** >** 2**. The calamenoic acid (**4**) showed comparatively better activity than the GHS. The IC_50_ values (Table 2) of calamenoic acid and GHS were 112.4 ± 29.3 *μ*M y 471 ± 77 *μ*M.

### 3.2. Evaluation of Anti-Inflammatory Effect

The results show that only the compounds** 2**,** 4** and** 16** displayed a significant anti-inflammatory activity, but this was less than that of indomethacin used as reference drug. The rest of the compounds showed a percentage of inhibition lower than 50%. (Table 3).

### 3.3. Life Span Assay

**Figure 3:**
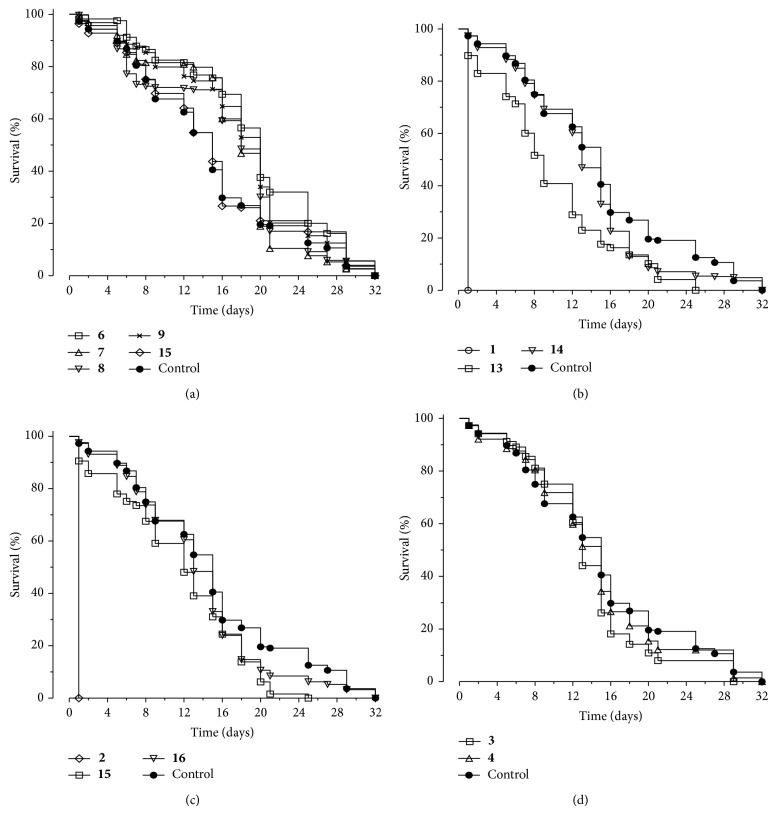
Lifespan survival curves in the presence* H. inuloides* metabolites. Labels denote the compound used (100 *μ*M). Curve (a) flavonoids, curves (b), (c), and (d) natural and semisynthetic cadinane type compounds.

Exposure of* C. elegans* to compounds gave the following results: at 100 *μ*M flavonoids type compounds extended half-life compared to the control (Table 4). Significant differences were observed for treatments compared with the vehicle-treated control (*P* < 0.001). The survival curve exhibited significant difference among worms fed with flavonoid compounds. There was not an increase in lifespan of the worm in the presence of cadinane compounds and some of them decreased survival as shown in Figure 3.

## 4. Discussion

*H. inuloides* metabolites showed to possess the ability to inhibit lipid peroxidation and scavenging free radicals and ROS. In lipid peroxidation assay we observed that the replacement of H at the hydroxyl in position 7 of sesquiterpenes** 1** and** 2** led to a decrease in the activity. It is further noted that the increase in the unsaturation of cadinanes led to a decrease in their activity, which is observed for compound** 18** whose activity is greater than** 1** that is greater than** 2**. In the case of flavonoid type compounds,** 6** (quercetin) had the best ability to inhibit lipid peroxidation; natural methoxy derivatives** 7** and** 8** showed lower activity. These results are consistent with those reported that indicate that methoxy substituent perturbs the planarity due to steric hindrance imparted by the methyl group [36]. It is reported that the antioxidant activity of flavonoids is proportional to the number of hydroxyl groups present in the molecule [37]. Compound** 6** (quercetin) having a 3,5,7,3′,4′-pentahydroxy group showed DPPH radical scavenging activity. Compounds** 1** and** 2** showed a statistically similar activity to** 6** and higher than that of *α*-tocopherol. We noted that several tested compounds had the ability to inhibit lipid peroxidation; however, few of them reduced the DPPH radical. The above observations may be explained because the DPPH radical has a long life, and no resemblance to the highly reactive peroxyl radicals involved in lipid peroxidation, and some antioxidants that react quickly with peroxyl radicals react slowly or are inert to DPPH [38].

Hypochlorous acid radicals have the ability to inactivate the antioxidant enzyme catalase through degrade its heme group [33]. Catalase inactivation is inhibited in the presence of some* H. inuloides* compounds. The results showed that compounds are more efficient scavenger than the standard ascorbic acid. Among the flavonoid type compounds,** 9** showed the highest ability to scavenge hypochlorous acid while** 7** and** 8** showed lower activity. In the last two compounds the hydroxyl at position 3, present in the quercetin molecule has been replaced by a methoxy group. It has been reported that the hydroxyl at position 3 of the C ring of quercetin plays an important role in the antioxidant activity including scavenging hypochlorite [39].

Hydroxyl free radicals cause damage to oxidative cells trough damage DNA, lipids, and proteins [31]. The flavonoids scavenge hydroxyl (OH^∙^) radicals. Hydroxyl radical (OH^∙^) generated by the Fenton reaction system was evaluated by TBARS assay. We observed that compound** 9** was more active than** 7**, which in turn was more active than** 8**, with only one hydroxyl group. The results obtained are consistent with reports indicating that the scavenging activity of flavonoids increases with the number of free hydroxyl groups, and it is independent of the presence of double bond between C-2 and C-3 of ring C [40].

In biological systems H_2_O_2_ produces hydroxyl ions and hydroxyl radicals by reacting with Fe^2+^ and Cu^2+^ ions and they have been related to the initiation of many toxic effects [41]. It is therefore biologically advantageous for cells to control the accumulation of H_2_O_2_.* H. inuloides* metabolites showed ability to trap peroxide; however, they were no better than pyruvate.

The superoxide radical is a highly reactive species and not readily diffuses through the cell. Because the main production site of O_2_^−^ is the inner mitochondrial membrane, it has been proposed that mitochondrial DNA (mtDNA) is the main target of damage [42]. We noted that compounds** 9** and** 7** have the capacity to scavenging superoxide. It has been reported that the superoxide scavenging capacity of flavonoids is dependent on the number of hydroxyl groups in the B ring, on the presence of a free hydroxyl group at C3, on the presence of a saturated C2-C3 bond, and the absence of a C4 carbonyl group [43]. Also it has been reported that the number of conjugated double bonds and the presence of conjugated keto groups increase the quenching rate, while the presence of a hydroxyl, epoxy, and methoxy group has lesser effects [44]. Cadinane type compound** 4** and flavonoid** 9** showed the best ability to scavenge the singlet oxygen ^1^O_2_; other flavonoid type compounds showed lesser activity. The ^1^O_2_ scavenger activity of flavonoids is associated with the presence of a catechol moiety on ring B and with the presence of a hydroxyl group activating the double bond on C ring [45]. The cadinane type sesquiterpenes showed capacity to trap singlet oxygen greater than glutathione. In another study the ability of 7-hydroxy-3,4-dihydrocadalene to trap this species has been reported [46].

Preparations from* H. inuloides* are used as anti-inflammatory and analgesic agents in Mexican traditional medicine, and these properties have been investigated in several studies [13, 47, 48]. The results of anti-inflammatory assay activity showed that acetylation of compounds** 1** and** 2** to the corresponding derivatives** 13** and** 15,** respectively, generated a change in biological response, since these derivatives exhibited proinflammatory activity. However, the benzoyl derivatives** 14 **and** 16** (obtained from** 1** and** 2**) resulted in opposite effects. Compound** 14** increased the anti-inflammatory activity with respect to** 1**, while compound** 16 **displayed less activity in comparison with** 2**. It has previously been reported that quercetin (**6**) showed anti-inflammatory capacity in the mice ear edema test [13]; in this study it was observed that naturally methylated derivative** 7** and semisynthetic acetylated derivative** 17** displayed little activity in this assay (see Table 3).

We evaluated the effect of some natural products isolated from* H. inuloides* and derivatives in extension of life of* C. elegans* nematode (Figure 3). Treatment of* C. elegans* with some* H. inuloides* metabolites prolonged the lifespan of the worm and the best effects were obtained with flavonoid type compounds. The results are consistent with other studies conducted wherein it has been reported that certain compounds of flavonoid type increase lifespan of* C. elegans*, which protect against oxidative stress and cause an increase in the translocation of the transcription factor DAF-16 [49]. In another study it was observed that blueberry polyphenols increased lifespan and slowed aging related decline in* C. elegans*, but these benefits did not just reflect antioxidant activity [50]. Similarly, results of Wilson et al. [51] showed that the prolongevity effect of myricetin is dependent on DAF-16 and not on direct antioxidative effects of the flavonoid. Büchter et al. [52] observed that quercetin significantly increased reproductive capacity of* C. elegans* and enlarged the body size, whereas no modification of these characteristics was induced by their methylated derivatives, isorhamnetin, and tamarixetin. Certain cadinane type compounds isolated from* H. inuloides* have shown to possess antioxidant activity, but its antioxidant capacity was not reflected in an increase in the life span of* C. elegans*. Although several compounds isolated from* H. inuloides* have antiantigiardial activity [21] and it has been found that structurally similar compounds showed activity against* Leishmania chagasi* promastigotes [53].

## 5. Conclusions

In the present paper, we have shown that* H. inuloides* metabolites in vitro scavenged to O_2_^−^, HOCl, H_2_O_2_, OH^∙^, and ^1^O_2_ in a concentration-dependent way. The IC_50_ in some cases were comparable to the reference compound. These observations suggest that metabolites of* H. inuloides* have the ability to capture free radicals. The results of the anti-inflammatory assay of the natural products and some derivatives showed both anti- and proinflammatory effects, depending on the functionalization of the OH^−^ groups. We have found that antioxidant capacities are not predictive of* C. elegans* lifespan benefits. Even where a life span extension and antioxidant effect were observed, it appears that other factors are also likely to be involved in modulating lifespan.

## Figures and Tables

**Table 1:** Capacity to inhibit lipid peroxidation and to reduce the DPPH radical.

Compound	IC_50_ *μ*M	
	Lipid peroxidation	Reduction of DPPH
**1**	4.72 ± 0.11	>100
**2**	3.09 ± 0.19	30.66 ± 8.14
**3**	nd^*^	nd
**4**	nd	nd
**6**	4.30 ± 0.27	13.11 ± 1.2
**7**	6.16 ± 0.06	>100
**8**	nd	nd
**9**	14.46 ± 0.61	6.97 ± 0.14
**11 **	17.35 ± 1.40	nd
**12**	nd	—
**13**	1.82 ± 0.12	nd
**14**	>100	nd
**15**	>100	nd
**16**	>100	nd
**17**	3.67 ± 0.43	—
**18**	**0.58** ± **0.008**	nd
***α*-Tocopherol**	6.78 ± 2.16	31.74 ± 1

^*^Not determined: at the concentrations tested the activity of compounds was low.

**Table 2:** Scavenging capacity of *H. inuloides *metabolites on hypochlorous acid, hydroxyl radical, peroxide, superoxide anion, and singlet oxygen (IC_50_).

Compound	Scavenging capacity IC_50_ *μ*M				
	HOCl	OH^•^	H_2_O_2_	O_2_^−^	^ 1^O_2_
**2**	69.1 ± 8.8^*^	133.4 ± 32.6	270 ± 2.7^*^	114.6 ± 13.9^*^	880.4 ± 55.4^*^
**3**	202.3 ± 21.9^*^	545.4 ± 23.0^*^	422 ± 8.0^*^	105 ± 7.2^*^	538.9 ± 58.6
**4**	203.5 ± 23.9^*^	607.9 ± 33.5^*^	536 ± 14^*^	148 ± 13.0^*^	112.4 ± 29.3^*^
**7**	68.1 ± 5.77^*^	161.2 ± 27.2	201 ± 6.7^*^	20 ± 0.27^*^	209.7 ± 44.5^*^
**8**	133.9 ± 9.0^*^	187.1 ± 44.6	252 ± 6.5^*^	70.4 ± 3.3^*^	237.8 ± 43.1^*^
**9**	60.0 ± 5.2^*^	139.5 ± 1.1	154 ± 6.3^*^	12.2 ± 0.22	314.4 ± 15.8
**13**	106.1 ± 4.4^*^	523.8 ± 20.4^*^	322 ± 21.0^*^	181.5 ± 29.8^*^	697 ± 187.0^*^
**15**	110.0 ± 15.8^*^	435.78 ± 82.0^*^	434.5 ± 23.3^*^	244.1 ± 78.0^*^	478.6 ± 44.7^*^

Reference compound	Ascorbic acid	DMTU	Pyruvate	NDGA	GSH
	278.6 ± 16.7	131.5 ± 3.6	32.2 ± 1.7	11.6 ± 0.13	471 ± 77

Data represent the mean ± SE of the three independent assays. The results were analyzed by ANOVA. The Dunnet's multiple comparison test was used to compare outcomes between experimental and control group. ^*^ *P* < 0.05 versus reference compound.

**Table 3:** Anti-inflammatory activities of additional *H. inuloides* metabolites.

Compound (1 *μ*mol/ear)	Edema	%
	(mg, average SE)	Inhibition
**Negative control**	12.07	—
**1**	10.50 ± 0.99	5.41
**2**	1.85 ± 0.52	83.33^**^
**4**	5.27 ± 0.70	65.73^**^
**5**	9.80 ± 0.36	7.98
**7**	7.30 ± 1.15	38.86^**^
**9**	7.57 ± 0.45	36.63^**^
**13**	11.87 ± 0.66	−11.42
**14**	11.07 ± 1.15	11.06
**15**	13.60 ± 0.70	−13.90
**16**	3.73 ± 1.62	66.44^**^
**17**	9.07 ± 0.70	19.46
**18**	13.17 ± 0.51	13.57
**Indomethacin (0.31** *μ * **mol)**		61.90^**^

The data represent the mean of three animals ± the standard error of the mean. All the compounds were tested at 1 *μ*mol/ear doses. The results were analyzed by ANOVA. Student's *t*-test was used to compare outcomes between experimental and control groups. ^*^ *P* < 0.05 versus reference drug.

**Table 4:** Summary of *Caenorhabditis elegans* life span.

Treatment	Adult life span, 20°C		(*n*)	*P* versus control
	Mean	CI 95%		(Log-rank)
**1**	1	0.171–1.962	449	<0.0001
**2**	1	0.169–1.964	479	<0.0001
**3**	13.661	13.13–14.209	513	<0.0001
**4**	14.391	13.776–15.006	557	0.1175
**6**	**19.2**	18.41–19.82	444	<0.0001
**7**	16.991	16.387–17.596	470	<0.0001
**8**	16.583	15.926–17.239	575	<0.0001
**9**	17.74	17.04–18.44	466	<0.0001
**13**	11.012	10.414–11.609	434	0.0080
**14**	13.424	12.845–14.003	531	0.0001
**15**	11.659	11.103–12.215	499	0.0150
**16**	13.520	12.917–14.123	496	0.0001
**17**	15.216	14.478–15.955	499	0.1751
**Control**	14.943	14.471–15.414	1098	
